# Studying the differential efficacy of postsymptom antitoxin treatment in type A versus type B botulism using a rabbit spirometry model

**DOI:** 10.1242/dmm.035089

**Published:** 2018-09-27

**Authors:** Amram Torgeman, Arieh Schwartz, Eran Diamant, Tzadok Baruchi, Eyal Dor, Alon Ben David, Avi Pass, Ada Barnea, Arnon Tal, Amir Rosner, Osnat Rosen, Ran Zichel

**Affiliations:** 1 Department of Biotechnology, Israel Institute for Biological Research, Ness Ziona 7410001, Israel; 2 The Veterinary Center for Pre-clinical Research, Israel Institute for Biological Research, Ness Ziona 7410001, Israel

**Keywords:** Antitoxin, Botulism, Efficacy, Spirometry, Postsymptom

## Abstract

Botulinum neurotoxin (BoNT) serotypes A, B and E are responsible for most cases of human botulism. The only approved therapy for botulism is antitoxin treatment administered to patients after symptom onset. However, a recent meta-analysis of antitoxin efficacy in human botulism cases over the past century concluded that a statistically significant reduction in mortality is associated with the use of type E and type A antitoxin, but not with type B antitoxin. Animal models could be highly valuable in studying postsymptom antitoxin efficacy (PSAE). However, the few attempts to evaluate PSAE in animals relied on subjective observations and showed ∼50% protection. Recently, we developed a novel spirometry model for the quantitative evaluation of PSAE in rabbits and used it to demonstrate full protection against BoNT/E. In the current study, a comparative evaluation of PSAE in botulism types A and B was conducted using this quantitative respiratory model. A lethal dose of each toxin induced a comparable course of disease both in terms of time to symptoms (TTS, 41.9±1.3 and 40.6±1.1 h, respectively) and of time to death (TTD, 71.3±3.1 and 66.3±1.7 h, respectively). However, in accordance with the differential serotypic PSAE observed in humans, postsymptom antitoxin treatment was fully effective only in BoNT/A-intoxicated rabbits. This serotypic divergence was reflected by a positive and statistically significant correlation between TTS and TTD in BoNT/A-intoxicated rabbits (r=0.91, *P*=0.0006), but not in those intoxicated with BoNT/B (r=0.06, *P*=0.88). The rabbit spirometry system might be useful in the evaluation toolkit of botulism therapeutics, including those under development and intended to act when antitoxin is no longer effective.

## Abstract (precis)

**Summary:** Here, we used a quantitative rabbit respiratory model to study the human-related, differential antitoxin efficacy observed in type A and type B botulism.

## INTRODUCTION

Botulinum neurotoxins (BoNTs) produced by the anaerobic bacterium *Clostridium botulinum* are the most potent toxins known in nature, with an estimated human lethal dose 50% (HLD_50_) of 1 ng/kg of body weight (Arnon et al., 2001). BoNT serotypes A, B, E and, rarely, F are responsible for most cases of human botulism (Pirazzini et al., 2017). Following entry into the circulation, BoNTs block acetylcholine transmission across neuromuscular junctions at presynaptic motor-neuron terminals and cause bilateral flaccid paralysis that eventually ends in respiratory failure (Dembek et al., 2007; Sugiyama, 1980). Widespread outbreaks of food-borne botulism might involve dozens of infected people who without adequate treatment could die (Kongsaengdao et al., 2006; McCarty et al., 2015; Weber et al., 1993); thus, BoNTs pose a significant concern for health authorities. In addition, owing to their extreme potency, BoNTs are classified as category A bio-threat agents (Centers for Disease Control and Prevention, https://emergency.cdc.gov/agent/agentlist-category.asp).

The only currently approved therapy for botulism is postsymptomatic administration of botulinum antitoxin and, in severe cases, intensive supportive care by means of mechanical ventilation. Antitoxin preparations are derived from equine plasma for adult patients (Centers for Disease Control and Prevention, 2010) or from human plasma in cases of infant botulism (http://www.infantbotulism.org). Botulinum antitoxin is expected to be useful mainly in neutralizing circulating BoNT molecules that are not yet bound to nerve endings (Sobel, 2005). Hence, prompt antitoxin treatment should slow the course of the disease and reduce pulmonary distress by preventing the toxin from binding to its target (Tacket et al., 1984). Indeed, data collected from observations in human clinical cases and from animal studies support the existence of a critical ‘therapeutic time window’ for effective antitoxin treatment following botulinum intoxication (Tacket et al., 1984). Notably, antitoxin is given only to symptomatic patients owing to concerns of potential adverse effects associated with its equine source and its immunogenic nature. Nevertheless, although antitoxin is administered to patients only after symptom onset, its efficacy evaluation in animal studies has been mostly related to time postintoxication, regardless of symptoms (Franz et al., 1993; Habermann and Bernath, 1975; Iida et al., 1970a,b; Lewis and Metzger, 1979; Ono et al., 1969, 1970).

Recently, we established a novel quantitative and objective animal model for the evaluation of postsymptom antitoxin efficacy (PSAE) that relies on spirometry detection of early respiratory symptoms of botulism in rabbits (Diamant et al., 2018). This model was used to demonstrate, for the first time, full protection of animals intoxicated with a lethal dose of BoNT/E, the fastest acting of all BoNT serotypes.

In humans, BoNT/A and BoNT/B intoxication pose a greater threat than BoNT/E for several reasons: they are more potent, have a longer *in vivo* activity resulting in extended hospitalization time and, at least for BoNT/A, are more frequently associated with human botulism (Johnson and Montecucco, 2008; Rossetto et al., 2014). Importantly, a recent meta-analysis of antitoxin efficacy in human cases of food-borne botulism over the past century concluded that a statistically significant reduction in botulism-related mortality was associated with the use of type E and type A antitoxins, but not with the use of type B antitoxin (O'Horo et al., 2018). As the rabbit spirometry model was the first to produce comparable postsymptom treatment outcomes to those obtained in human patients, we sought to use this model in the current study to conduct a comparative evaluation of PSAE in type A and type B botulism. BoNT/A and BoNT/B intoxications induced a similar course of disease both in terms of time to symptoms and time to death. Nevertheless, in agreement with the reported data from human clinical cases, complete protection could be demonstrated only for type A botulism. This serotypic divergence in responsiveness to antitoxin treatment is discussed in relation to the similar pattern observed in human clinical cases. A potential mechanism of the phenomenon that stems from the differential catalytic efficiencies of BoNT serotypes along with distribution variances of neural receptors and intracellular components involved in the mechanism of action of botulinum toxins in vertebrates is suggested.

## RESULTS

### Reduced minute volume is a cross-serotypic early symptom of botulism in rabbits

**Fig. 1.**
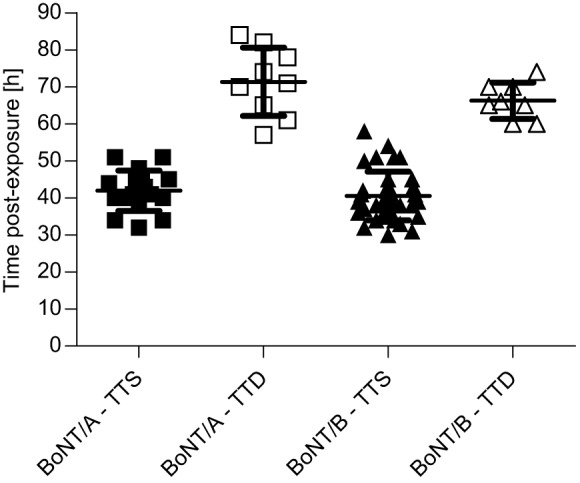
**Comparable course of disease in rabbits** **intoxicated with BoNT/A or BoNT/B.** Rabbits were exposed to 4 RbIMLD_50_ of BoNT/A or BoNT/B and the time from intoxication to symptom onset and death was individually determined. TTS was determined for each rabbit based on the MV parameter. TTS data were collected from negative control (toxin only) and from antitoxin-treated rabbits. The TTD data are from toxicity experiments. Data represent mean±s.e.m.

The rabbit spirometry model was previously used to quantify early respiratory symptoms of type E botulism, which then served as a clinical trigger to treat (Diamant et al., 2018). The goal of the current study was to evaluate anti-BoNT/A and anti-BoNT/B antitoxin efficacy after the onset of respiratory botulism symptoms using the spirometry model. To this end, the toxicity of BoNT/A and BoNT/B in rabbits was first studied by the determination of intramuscular LD_50_. A dose of 1 intramuscular rabbit LD_50_ (1 RbIMLD_50_) of BoNT/A or BoNT/B was found to be equivalent to 4 and 5 mouse intraperitoneal LD_50_ per kg (MsIPLD_50_/kg), respectively. To allow characterization of the respiration physiology in botulinum-intoxicated rabbits, animals were exposed to 4 RbIMLD_50_ of BoNT/A or BoNT/B and spirometry parameters were monitored pre- and postexposure. Values were determined individually for all tested rabbits, as previously described (Diamant et al., 2018), and were scored according to time to symptoms (TTS), i.e. the time postexposure at which a statistically significant deviation (mean minus 2 s.d.) from the pre-exposure limit had occurred. In accordance with the results obtained for BoNT/E, the earliest spirometry parameter deviation in both BoNT/A- and BoNT/B-intoxicated rabbits was observed in the minute volume (MV) parameter (TTS of 41.9±1.3 h and 40.6±1.1 h for BoNT/A and BoNT/B, respectively; Fig. 1). MV was also the most consistent parameter with relative standard deviation values of 13% and 16% for BoNT/A and BoNT/B, respectively. Thus, the MV parameter was chosen to become the leading clinical symptom for investigating antitoxin efficacy in symptomatic rabbits for both toxin serotypes. As with TTS, examination of time to death (TTD) after BoNT/A and BoNT/B exposure revealed comparable values (71.3±3.1 h and 66.3±1.7 h, respectively) (Fig. 1). Thus, considering the similarity of TTS average values, both toxins induced a comparable course of disease.

### Administration of antitoxin after the onset of respiratory symptoms fully protects

### rabbits intoxicated with BoNT/A, but not those intoxicated with BoNT/B

**Fig. 2.**
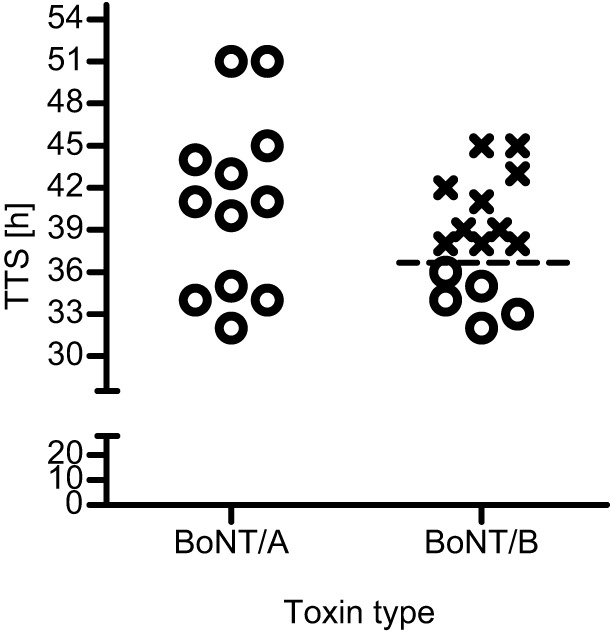
**Postsymptom antitoxin treatment is fully protective in rabbits** **intoxicated with BoNT/A, but not in those intoxicated with BoNT/B.** Rabbits were exposed to 4 RbIMLD_50_ of BoNT/A or BoNT/B, and antitoxin (215 IU/kg) was administered intravenously immediately after the onset of spirometry symptoms. Each plotted symbol (o, survival; x, death) refers to an individual animal. The dashed line represents the latest time point (36 h) at which symptomatic rabbits were fully protected by antitoxin treatment. Data were collected from at least three independent experiments. Each experiment included negative control (toxin only) rabbits. TTS values of accompanying negative control (toxin only) rabbits (*n*=8, 42.25±1.19 h and 41.75±1.32 h for BoNT/A and BoNT/B, respectively) were comparable to those of antitoxin-treated rabbits.

To evaluate antitoxin efficacy in a clinically relevant fashion, spirometry was monitored before and after the administration of 4 RbIMLD_50_ of BoNT/A or BoNT/B to groups of 12-15 rabbits. Rabbits were treated intravenously with 215 international units per kg (IU/kg) of antitoxin A or B immediately after the onset of spirometry symptoms (Fig. 2). As shown in Fig. 2, symptom onset after BoNT/A or BoNT/B exposure ranged similarly (TTS of 32-51 h and 32-45 h, respectively). Nevertheless, their immediate postsymptom treatment outcome was essentially different. BoNT/A antitoxin therapy given immediately after symptom onset was fully protective, as all treated rabbits survived (12/12) even when symptom onset was measured as late as 51 h. Moreover, rabbits (*n*=2) injected with an elevated BoNT/A dose (8 RbIMLD_50_) and treated with the same dose of antitoxin immediately after symptom onset were also fully protected. In all treated rabbits, MV gradually returned to normal values and full respiration recovery was retained. In contrast to the results obtained with BoNT/A, PSAE in rabbits intoxicated with BoNT/B was limited and of a time-dependent nature. Although all rabbits treated with antitoxin up to 36 h postexposure were fully protected by BoNT/B antitoxin, no protection was observed when treatment was administered beyond this time point (Fig. 2). Notably, TTD of antitoxin-treated rabbits (86.3±9.7 h; Fig. 2) was significantly prolonged compared with that of negative control (toxin only) rabbits (66.3±1.7 h; Fig. 1, *P*=0.03).

To further establish the time-dependent PSAE observed in BoNT/B-intoxicated rabbits, an additional set of eight rabbits were exposed to 4 RbIMLD_50_ of BoNT/B and were divided into four pairs according to postexposure time treatment. Each pair was treated with BoNT/B antitoxin, regardless of the time of symptom onset, at four postexposure time intervals (30, 33, 36 and 39 h). All rabbits treated before and up to 36 h postexposure were protected, whereas one of two rabbits treated at 39 h survived (Table 1). Notably, the rabbit that succumbed to the toxin did not show a significant spirometry symptom up until the time of antitoxin administration, supporting the view of a time course-related antitoxin efficacy. The survival of one rabbit at 39 h might be a consequence of normal animal variance. Nevertheless, taken together with the ten nonsurviving treated rabbits in the experiment presented in Fig. 2, the beneficial response of antitoxin treatment given after 36 h of BoNT/B intoxication was less than 10%.

**Table 1.**
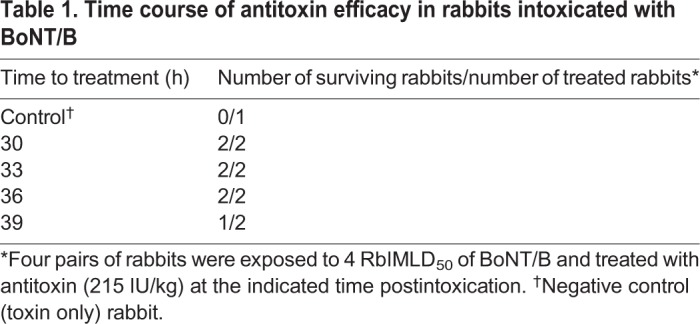
**Time course of antitoxin efficacy in rabbits intoxicated with BoNT/B**

### Positive and significant correlation between TTS and TTD in rabbits intoxicated with BoNT/A, but not in those intoxicated with BoNT/B

**Fig. 3.**
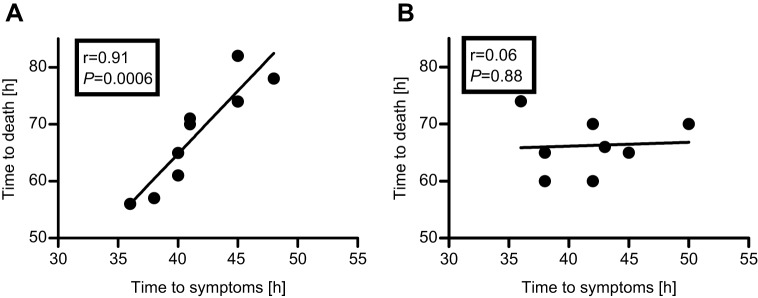
**Correlation analysis between TTD and TTS in rabbits intoxicated with a lethal dose of BoNT/A or BoNT/B.** (A,B) Rabbits were exposed to 4 RbIMLD_50_ of BoNT/A (A) or BoNT/B (B) and the correlation between TTD and TTS was analyzed. The correlation is based on negative control (toxin only) data presented in Fig. 1.

In our previous study, a positive and statistically significant correlation between TTS and TTD was found in BoNT/E-intoxicated rabbits, and all animals treated with antitoxin immediately following symptom onset survived (Diamant et al., 2018). In an attempt to determine whether the differential outcome of postsymptom antitoxin treatment in BoNT/A- and BoNT/B-intoxicated animals is related to TTS-TTD association, the correlation between these two parameters was analyzed for each of the two toxins. Evidently, a dramatic and significantly different pattern of correlation results was observed. Although the TTD-TTS correlation was positive and statistically significant for BoNT/A (r=0.91, *P*=0.0006; Fig. 3A), a low and insignificant correlation was found for BoNT/B (r=0.06, *P*=0.88; Fig. 3B).

### Efficacy of delayed postsymptom antitoxin treatment in BoNT/A-intoxicated rabbits

**Fig. 4.**
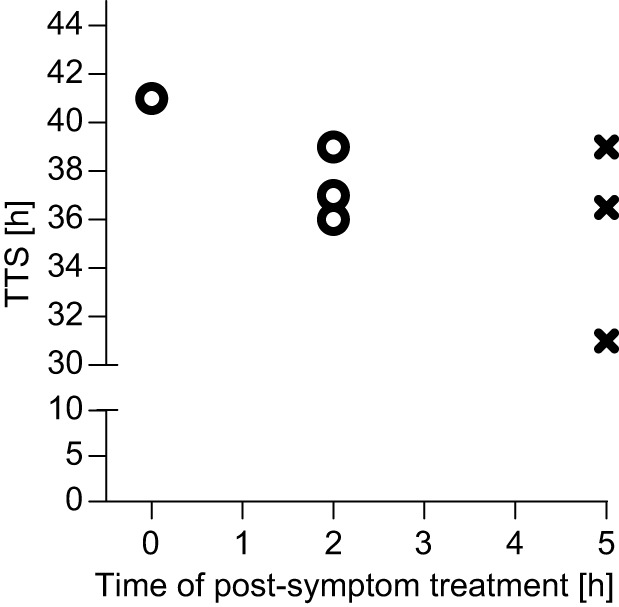
**Delayed antitoxin treatment following botulism A symptom onset.** Rabbits were intoxicated with 4 RbIMLD_50_ of BoNT/A and treated with 215 IU/kg of antitoxin immediately (0 h), 2 h or 5 h postsymptom onset. Each plotted symbol (o, survival; x, death) refers to an individual animal. The TTS of the accompanying negative control (toxin only) rabbit (*n*=1) was 36 h.

Clinical diagnosis of botulism in human patients usually takes days to be completed (Hellmich et al., 2018). This further delays the administration of antitoxin to patients who are already symptomatic upon arrival at the clinic. Therefore, we aimed to define the therapeutic time window for effective postsymptom antitoxin treatment in our system. To this end, antitoxin was administered to BoNT/A-intoxicated rabbits 2 h or 5 h postsymptom onset and their survival was monitored (Fig. 4). The control rabbit (*n*=1) that received antitoxin immediately after the onset of the respiratory symptoms survived the 4 RbIMLD_50_ BoNT/A intoxication (Fig. 4). Likewise, all rabbits (*n*=3) survived when antitoxin treatment was given 2 h postsymptom onset (Fig. 4). Further delaying the treatment to 5 h postsymptom onset, however, resulted in a lack of protection (*n*=3). These data emphasize how tightly the respiratory symptoms are related to the clinical course of type A botulism in our model. Immediate antitoxin treatment provided full protection even when symptom onset was the latest to be observed (TTS=41 h; Fig. 4) or as late as 51 h (Fig. 2), whereas treatment delayed by 5 h postsymptoms was ineffective even when symptoms appeared as early as 31 h. This observation clearly validates the relevance of the respiratory symptoms as an adequate clinical symptom in relation to botulism A disease in rabbits.

## DISCUSSION

In most reported botulism animal studies aimed at evaluating postexposure treatment efficacy, antitoxin was administered in a time postexposure mode. Only a few attempts have been made to study PSAE (Adler and Franz, 2016; Dack and Wood, 1928; Kodihalli et al., 2017; Oberst et al., 1968; Food and Drug Administration BAT^®^ Data Sheet, https://www.fda.gov/downloads/biologicsbloodvaccines/bloodbloodproducts/approvedproducts/licensedproductsblas/fractionatedplasmaproducts/ucm345147.pdf). Moreover, symptoms in these studies were limited to those that can only be noticed by simple observation. Notably, the detection of observed symptoms in animals is subjective and can be difficult to determine. In addition, human patients report early botulism symptoms such as blurred vision, dry mouth and diplopia or just general feelings of being unwell long before the appearance of observable signs, such as ptosis and difficulty in speaking (Mottate et al., 2016). By contrast, animals, especially rodents, do not present such facial symptoms and obviously cannot report their situation (Hellmich et al., 2018). Therefore, observed early symptoms in animals might be considered as appearing at a later stage of the disease than early symptoms in humans. These factors could explain, at least partially, the reason for the limited success of PSAE studies in animal models to date.

In the current study, we have used an objective and quantitative respiratory symptom to evaluate antitoxin efficacy in symptomatic rabbits. Symptom onset in this system appeared earlier than any visible symptom detectable by the naked eye. The validity of this system for early diagnosis of botulism was recently established by demonstrating full protection of symptomatic rabbits previously exposed to a lethal dose of BoNT/E, the fastest-acting toxin of all BoNT serotypes. Several respiratory parameters were screened in the search for an optimal symptom for botulism during the establishment of the spirometry model. Taken together, the data collected here and in our previous study showed that the MV parameter presents the lowest variance and is the earliest to appear in rabbits exposed to all three botulinum serotypes, which are responsible for >99% of human cases of botulism (Diamant et al., 2018).

Rabbits exposed to BoNT/A or BoNT/B presented a similar course of disease in terms of TTS and TTD (Fig. 1). Moreover, the toxicity values measured for both serotypes were almost identical. In a previous study, we found that TTS and TTD were significantly shorter in rabbits exposed to an even lower lethal dose of BoNT/E (Diamant et al., 2018). This serotypic divergence in the dynamic of the disease was reported previously (Wang et al., 2008; Woodruff et al., 1992). Indeed, individuals who were exposed to BoNT/E in food were admitted to hospital as early as 24 h after ingestion of contaminated food, whereas in cases of type A botulism the average time for hospitalization can vary up to 7 days postexposure (Woodruff et al., 1992).

In the current study, all rabbits exposed to a lethal dose of BoNT/A (4 IMRLD_50_) and treated with a human dose (body weight normalized) of antitoxin after the onset of botulism symptoms survived. Previous studies of PSAE in BoNT/A-intoxicated animals showed mostly limited efficacy (Adler and Franz, 2016; Kodihalli et al., 2017; Food and Drug Administration BAT^®^ Data Sheet, https://www.fda.gov/downloads/biologicsbloodvaccines/bloodbloodproducts/approvedproducts/licensedproductsblas/fractionatedplasmaproducts/ucm345147.pdf). Adler and Franz reported on the efficacy of equine F(ab′)_2_ antitoxin administered either 24 h postexposure or after the onset of observed symptoms in nonhuman primates exposed to a lethal dose of inhalational BoNT/A (36 LD_50_). Although all animals (4/4) treated with 14 IU/kg of antitoxin 24 h postexposure survived, no survival (0/4) was recorded when antitoxin was administered after the onset of first signs of intoxication, even at a dose as high as 429 IU/kg (Adler and Franz, 2016). In a more recent rhesus macaque study, treatment with equine heptavalent antitoxin after the onset of clinical signs following intravenous exposure to BoNT/A (1.7 LD_50_) conferred only partial protection (46% survival) (Kodihalli et al., 2017). In small rodents, a higher PSAE was reported (Mimran et al., 2012; US Food and Drug Administration BAT^®^ Data Sheet, https://www.fda.gov/downloads/biologicsbloodvaccines/bloodbloodproducts/approvedproducts/licensedproductsblas/fractionatedplasmaproducts/ucm345147.pdf). The considerably improved antitoxin efficacy obtained using the rabbit spirometry system in the current study might be attributed to its sensitivity and to the quantitative and objective nature of the spirometry symptoms that appeared hours before any visible sign of intoxication could be observed.

Despite having very similar TTS and TTD values, PSAE dramatically differed between BoNT/A- and BoNT/B-intoxicated rabbits. Although PSAE was fully protective in BoNT/A-intoxicated animals, only partial protection was obtained in rabbits exposed to BoNT/B. This serotypic divergence in PSAE closely co-aligned with the efficacy pattern observed in humans, as recently reported in a meta-analysis of antitoxin efficacy in food-borne botulism cases covering nearly a century (O'Horo et al., 2018). The study concluded that a significant reduction in botulism-related mortality is associated with the use of type E and type A antitoxin, but not with type B antitoxin. The ability to monitor individual TTS and TTD in our study in a highly controlled experiment demonstrated that PSAE is tightly associated with the correlation between these two fundamental characteristics of the disease. A high PSAE was associated with a positive and statistically significant correlation between TTS and TTD (type A and type E botulism), whereas poor PSAE was associated with a lack of correlation between these two parameters (type B botulism).

The poor PSAE observed exclusively in BoNT/B-intoxicated rabbits suggests that at the time of symptom onset a lethal dose of BoNT/B has already entered the neurons, whereas considerable amounts of BoNT/A and BoNT/E were still circulating and available for neutralization. Such a phenomenon could occur as a consequence of differences in the endopeptidase catalytic efficiencies of the BoNT serotypes. Indeed, in studies employing the light chain (LC) of BoNT, Barbieri and colleagues reported the catalytic constant (*k*_cat_) of LC/A and LC/E for recombinant, soluble and membrane-free substrates to be 60 s^−1^ and 77 s^−1^, respectively, whereas that of LC/B was 1.08 s^−1^ (Chen and Barbieri, 2009; Chen et al., 2008, 2007). In addition, the catalytic efficiency (*k*_cat_/*K*_M_) of LC/B was shown to be an order of magnitude lower than that of LC/A and two orders of magnitude lower than that of LC/E (0.67, 3.7 and 50 s^−1^ µM^−1^, respectively). Thus, if the BoNT/B catalytic activity is significantly slower than that of BoNT/A and BoNT/E, the interval between cell entrance and neuron paralysis is prolonged and more BoNT/B molecules could intoxicate additional neurons before symptom onset. The similar TTS we observed in BoNT/A- and BoNT/B-intoxicated rabbits might suggest that the slower catalytic activity of BoNT/B was compensated for by a faster binding and internalization. In this case, it is reasonable to assume that at the time of symptom onset a significantly lower amount of BoNT/B was available in the circulation for neutralization by antitoxin and a lethal dose had already entered the neurons.

It should be noted that besides the catalytic efficiency, differences in the binding and catalytic target molecules of the BoNT serotypes might also contribute to the differential PSAE. Both BoNT/A and BoNT/E share common molecular characteristics that differ from those of BoNT/B. BoNT/A and BoNT/E bind the synaptic vesicle protein 2 (SV2) receptor on neural cells, whereas BoNT/B binds synaptotagmin (Syt) I and II. Moreover, BoNT/B differs from BoNT/A and BoNT/E in the SNARE target protein that is cleaved within the neural cells. BoNT/A and BoNT/E cleave the 25 kDa synaptosomal-associated protein (SNAP-25), whereas BoNT/B cleaves the vesicle-associated membrane protein (VAMP) (Arsenault et al., 2014; Binz et al., 1994; Kalandakanond and Coffield, 2001; Osen-Sand et al., 1996; Rhee et al., 1997; Schiavo et al., 1994, 1993a,b; Washbourne et al., 1997; Yadirgi et al., 2017; Yamamoto et al., 2012). In addition, variation in the presence, amounts, affinity, distribution and function of these receptors and SNARE proteins in the target tissues (Fox and Sanes, 2007; Peng et al., 2014) should also be considered as potential contributors to the differential PSAE after BoNT/B versus BoNT/A and BoNT/E intoxication.

In conclusion, the rabbit spirometry system was applied in the current work to study the efficacy of botulinum antitoxin, the only approved therapy against botulism, using the relevant trigger to treat in humans (i.e. postsymptom manifestation). The similar serotypic pattern of PSAE obtained in the current study and its correlation with reported human cases could emphasize the validity of the rabbit spirometry system and its superiority over other small animal models. Moreover, in respect to the animal rule (Food and Drug Administration, https://www.fda.gov/downloads/drugs/guidances/ucm399217.pdf), the serotypic divergence found in the current study suggests that a certain trigger to treat might not necessarily be valid for all BoNT serotypes in a given animal model. The rabbit spirometry system should be considered as a potential tool for the evaluation of new therapeutics, including drugs intended to act intracellularly at later stages of botulism where antitoxin is no longer effective.

## MATERIALS AND METHODS

### Ethics statement

All animal experiments were performed in accordance with Israeli law and were approved by the Ethics Committee for animal experiments at the Israel Institute for Biological Research (protocols RB-18-11, RB-24-11, RB-28-11, RB-12-12 and RB-14-12). All efforts were made to minimize animal suffering.

### Bacteria and toxins

*Clostridium botulinum* A and B strains were obtained from the Israel Institute for Biological Research (IIBR) collection (A198 and B592, respectively). A sequence analysis revealed compliance of the neurotoxin genes with serotypes 62A (GenBank accession number M30196) and Danish (GenBank accession number M81186) of *C. botulinum* types A1 and B1, respectively (Binz et al., 1990; Whelan et al., 1992). Toxin complexes were prepared from concentrated supernatants of cultures grown for 6 days in anaerobic culture tubes. The specific activity of the toxin preparations was determined to be 7.4×10^6^ MsLD_50_/mg and 1.5×10^7^ MsLD_50_/mg for BoNT/A and BoNT/B, respectively. Toxin stock was kept in 50 mM citrate buffer (pH 5.5) at −70°C. For determination of the lethal dose (mouse lethality assay), serial dilutions of the toxin in gelatin buffer (0.2% w/v gelatin in phosphate buffer pH 6.4) were injected intraperitoneally into groups of mice (*n*=5), as described by Malizio et al. (2000), and lethality was calculated according to the Spearman-Karber method (Irwin and Cheeseman, 1939).

### Antitoxin

Horse anti-BoNT/A or anti-BoNT/B plasma were collected from hyperimmune animals immunized with toxoid prepared by dialyzing the toxin complex against 0.14% formalin at 35°C for 2 weeks (Diamant et al., 2014). The Fc fragment was removed by pepsin digestion (Torgeman et al., 2017) and purified F(ab)′_2_ antitoxin-neutralizing activity was determined according to the European Pharmacopeia (European Directorate for the Quality of Medicines and Healthcare, 2014). Briefly, serial 1.2-fold dilutions of each antitoxin preparation were prepared. Simultaneously, a standard antitoxin preparation (calibrated according to the World Health Organization international standard antitoxin) was diluted to the final concentrations of 0.08, 0.10, 0.12 and 0.14 IU/ml. All antitoxin dilutions were then mixed with a fixed toxin test dose, and the mixtures incubated for 1 h at 25°C. Each mixture was injected intraperitoneally into four mice (CD-1; Charles River UK; 1 ml per mouse) and survival was monitored for 4 days. Antitoxin potency was calculated based on the lowest dilution of antitoxin that failed to protect the animals when compared with that of the standard antitoxin.

### Spirometry system

Inhalation parameters were recorded by a computer-monitored spirometry system. The system consisted of a snout-only mask connected to a T-type nonrebreathing two-way valve. A model 4100 Thermal Mass Flowmeter (TSI Inc., Shoreview, MN, USA) with low flow resistance was connected to the inhalation port of the valve. During measurements, non-anesthetized rabbits respired freely. The data collected from naive animals were in line with published values of normal rabbit respiration physiology (Bide et al., 2000).

Inhalation data were collected at 20 ms intervals (500 data points in 10 s). Each rabbit measurement lasted up to 2 min. Spirometry parameters that included MV, rate and tidal volume were measured and data were analyzed with Microsoft Excel (2013). On the basis of preliminary experiments, rabbit spirometry measurements conducted at intervals not shorter than 2 h did not affect spirometry performance. Accordingly, at least 12 independent measurements collected over 7 days (up to twice a day) were used to calculate the individual mean and s.d. of each one of the three spirometry parameters. Confidence limits were determined as the mean±2 s.d. (Diamant et al., 2018). Values below the lower limit in BoNT-exposed rabbits were attributed to breathing distress owing to the intoxication, and thus were considered as clinical symptoms of botulism.

### Antitoxin efficacy studies

Naive rabbits, previously monitored for individual respiratory performance, were injected in the quadriceps musculature of the hindlimb with 4 RbIMLD_50_ of BoNT/A or BoNT/B in 0.5 ml of gelatin-phosphate buffer (50 mM Na-phosphate, 0.2% gelatin, pH 6.5). Where indicated, BoNT/A was tested with 8 RbIMLD_50_. Rabbit spirometry was monitored starting at 24 h postintoxication and then at 2-24 h intervals. An animal that presented a significant deviation from its pre-intoxication respiration performance (lower than the mean value minus 2 s.d.) was defined as symptomatic. Antitoxin was intravenously administered to symptomatic rabbits at the indicated time postsymptom onset. The antitoxin dose was the equivalent in body weight to the human indicated dose (215 IU/kg). All experiments included negative control (toxin only) rabbits.
